# Pulmonary tumor thrombotic microangiopathy successfully treated with corticosteroids: a case report

**DOI:** 10.1186/s13256-017-1524-8

**Published:** 2017-12-23

**Authors:** Shinichi Miyazaki, Takuya Ikeda, Genshi Ito, Masahide Inoue, Keiji Nara, Yuko Nishinaga, Yoshinori Hasegawa

**Affiliations:** 1 Department of Respiratory Medicine, Yokkaichi Municipal Hospital, Sibata2-2-37, Shibata, Yokkaichi-shi, Mie 510-0822 Japan; 2 Department of Pathology, Yokkaichi Municipal Hospital, Sibata2-2-37, Shibata, Yokkaichi-shi, Mie 510-0822 Japan; 3 Department of Respiratory Medicine, Nagoya University Graduate School of Medicine, 65 Tsurumai-cho, Showa-ku, Nagoya, 466-8560 Japan

**Keywords:** Pulmonary embolism, Pulmonary hypertension, Rare lung diseases

## Abstract

**Background:**

Pulmonary tumor thrombotic microangiopathy is a special type of tumor thromboembolism. We report the case of a patient who developed pulmonary tumor thrombotic microangiopathy with alveolar hemorrhage. Almost all patients with pulmonary tumor thrombotic microangiopathy die within 1 week of the onset of dyspnea; however, the prognosis in this case was better, with 10 weeks of survival from presentation.

**Case presentation:**

A 62-year-old Japanese man was referred to our hospital with a 4-week history of dyspnea on exertion and severe pulmonary hypertension. Five years previously, he had undergone distal gastrectomy for gastric cancer. He was afebrile, normotensive, and hypoxemic. A physical examination was unremarkable except for purpura on his upper extremities and trunk. Blood tests showed anemia and disseminated intravascular coagulation. Chest computed tomography revealed diffuse ground-glass opacities with emphysema in his upper lungs, moderate pleural effusions, mediastinal lymphadenopathy, and enlargement of the right ventricle and main pulmonary artery. A computed tomography pulmonary angiogram showed no evidence of pulmonary embolism. Lung perfusion scintigraphy showed multiple segmental defects. Although recurrence of gastric cancer was confirmed from the results of bone marrow biopsy, bronchoscopy was not performed due to bleeding diathesis. He was treated with corticosteroids, antibiotics, and platelet transfusion, following which resolution of the abnormal lung shadows and right ventricular pressure overload along with partial alleviation of respiratory failure was observed. Because of his poor performance status, he was eventually transited to palliative care and died 6 weeks after admission. Necropsy of the lung confirmed the diagnosis of pulmonary tumor thrombotic microangiopathy with alveolar hemorrhage.

**Conclusions:**

Pulmonary tumor thrombotic microangiopathy should be considered in the differential diagnosis of patients with cancer who present with severe pulmonary hypertension. In pulmonary tumor thrombotic microangiopathy, local inflammation in pulmonary microvasculature may contribute to pulmonary hypertension, and regulation of inflammation using corticosteroids may help improve the prognosis.

## Background

Pulmonary tumor thrombotic microangiopathy (PTTM) is a special type of tumor thromboembolism. The condition is histologically characterized by microscopic tumor emboli with occlusive fibrointimal proliferation in the pulmonary microcirculation. It should be considered in the differential diagnosis of pulmonary hypertension, particularly in patients with known cancer. We report the case of a patient who developed PTTM with alveolar hemorrhage. Almost all patients with PTTM die within 1 week of the onset of dyspnea; however, the prognosis in this case was better, with 10 weeks of survival from the day of presentation.

## Case presentation

**Fig. 1:**
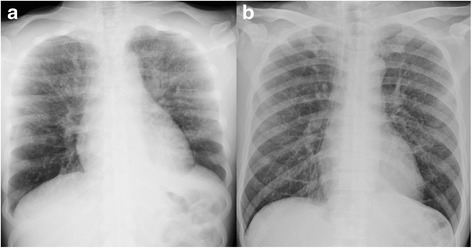
Chest X-ray. Chest X-ray at admission showing consolidation in the upper lungs (**a**); the abnormal shadow disappeared 4 weeks after admission (**b**)

A 62-year-old Japanese man presented with a 4-week history of dyspnea on exertion. There was no history of fever, anorexia, cough, or chest pain. He had visited another hospital, where he was found to be hypoxemic; a chest X-ray showed consolidation in his upper lungs (Fig. 1a). Echocardiography revealed marked right ventricular dilation and flattening of the interventricular septum with severe pulmonary hypertension (transtricuspid pressure gradient, 109 mm Hg). He was referred to our hospital within a few hours of presentation. Five years previously, he was diagnosed as having gastric cancer (pT2N2M0 stage IIIA), for which he underwent distal gastrectomy and adjuvant chemotherapy with tegafur plus uracil. On follow-up, after completion of 2 years of chemotherapy, no recurrence was observed. His family history was unremarkable. He was a former tobacco smoker with 20 pack-years of smoking history; there was no history of alcohol intake.

**Fig. 2:**
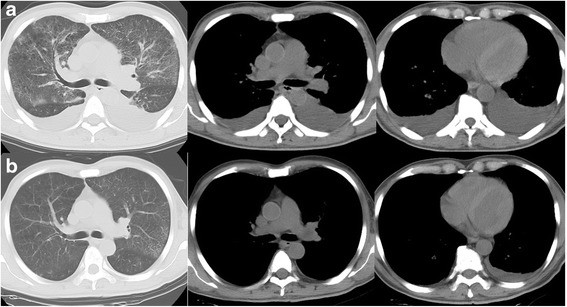
Computed tomography of the chest. **a** Computed tomography radiograph showing ground-glass opacities in the upper lungs with moderate pleural effusions, and findings suggestive of right ventricular pressure overload (enlargement of the right ventricle and main pulmonary artery). **b** Computed tomography radiograph at 4 weeks after admission showing resolution of the abnormal lung shadows along with signs of alleviation of the right ventricular pressure overload. These helical computed tomography images consisted of 5-mm collimation sections, with window settings appropriate for viewing both the lung (window level from −700 HU, window width from 2000 HU) and the mediastinum (window level from 40 HU, window width from 400 HU)

**Fig. 3:**
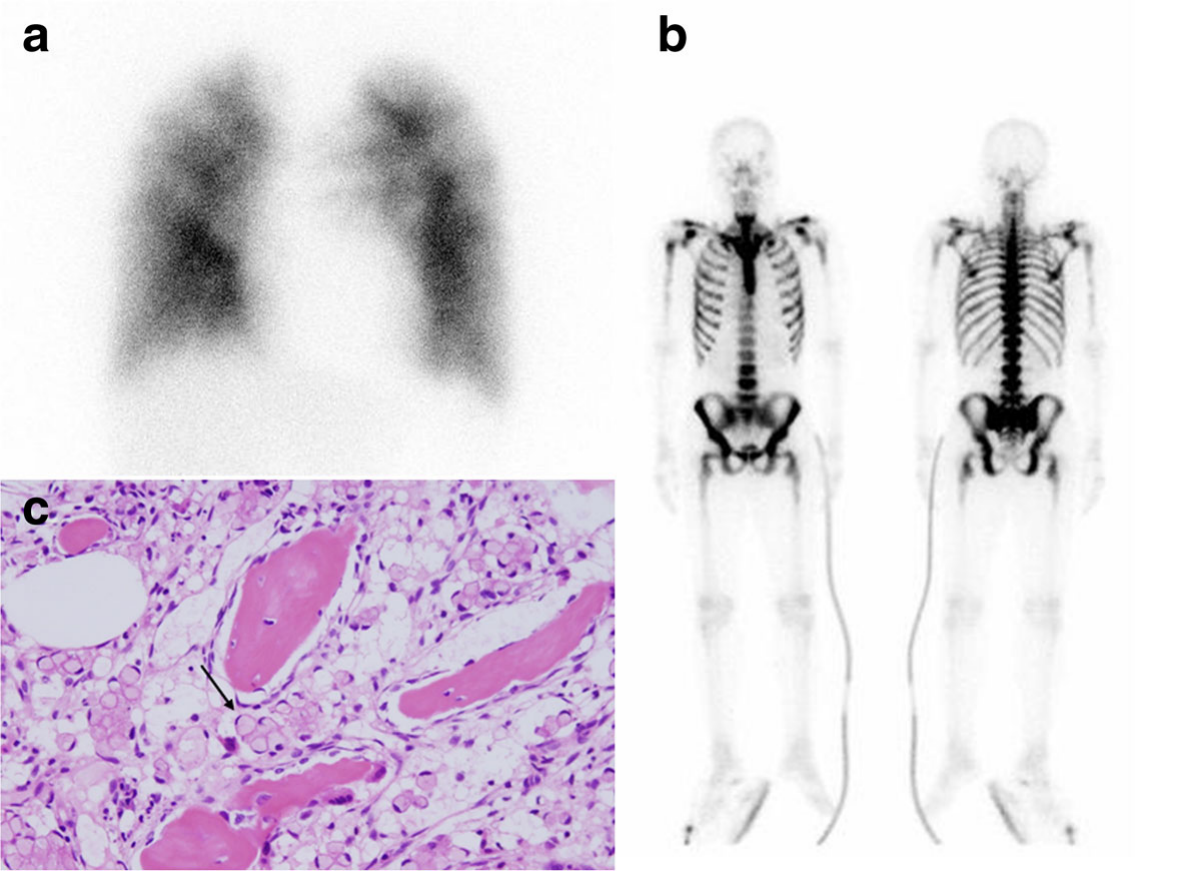
Lung perfusion scintigraphy, bone scintigraphy, and bone marrow biopsy. **a** Lung perfusion scintigraphy showing multiple segmental defects. **b** Bone scintigraphy showing diffuse uptake. **c** Signet ring cells (arrow) in bone marrow (hematoxylin and eosin, ×100)

He was afebrile, normotensive, and hypoxemic: blood oxygen saturation (SpO_2_) 88%, 3 L oxygen by nasal cannula. A physical examination was unremarkable with the exception of purpura on his upper extremities and trunk. Blood tests showed normocytic anemia with leukoerythroblastosis and disseminated intravascular coagulation. His serum alkaline phosphatase level was remarkably elevated (3205 U/L), but serum transaminase level was normal. Chest computed tomography (CT) revealed diffuse ground-glass opacities with emphysema in his upper lungs, moderate pleural effusions, mediastinal lymphadenopathy, and enlargement of the right ventricle and main pulmonary artery (Fig. 2a). A CT pulmonary angiogram (CTPA) showed no evidence of pulmonary embolism. Lung perfusion scintigraphy showed multiple segmental defects (Fig. 3a); bone scintigraphy showed diffuse uptake (Fig. 3b). Although recurrence of gastric cancer was confirmed from the results of a bone marrow biopsy (Fig. 3c), bronchoscopy was not performed due to bleeding diathesis.

In a patient with cancer presenting with subacute dyspnea, severe pulmonary hypertension and negative pulmonary angiogram are consistent with a diagnosis of PTTM. Differential diagnosis of diffuse ground-glass opacities in the upper lungs includes community-acquired pneumonia, interstitial pneumonia, and alveolar hemorrhage.

**Fig. 4:**
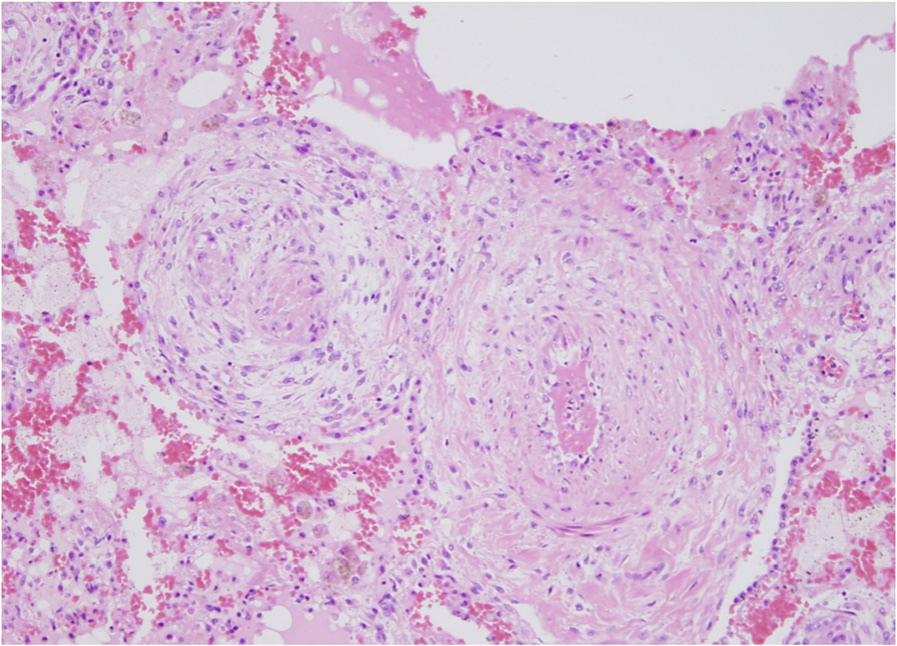
Necropsy of the lung. Obliterated small vessels with fibrointimal proliferation (hematoxylin and eosin, ×100)

He was treated with corticosteroids (prednisone 40 mg daily), intravenously administered antibiotics (cefepime), and platelet transfusion (three units of whole blood-derived platelets to maintain a platelet count of 50,000/μL). Following treatment, resolution of the abnormal lung shadows was observed along with improvement in right ventricular pressure overload (Figs. 1b and 2b), and partial alleviation of respiratory failure (SpO_2_ 96%, 2 L oxygen by nasal cannula). Plans for chemotherapy were deferred because of his poor performance status, and he was eventually transited to palliative care. Favorable conditions continued for several weeks. On day 41 of hospitalization, he developed fever with rigors, and empirical antibiotic treatment (cefepime and vancomycin) was initiated. He soon turned afebrile; however, on day 45 of hospitalization, he suddenly died of respiratory failure. Necropsy of the lung confirmed the diagnosis of PTTM (Fig. 4); there was no evidence of pulmonary embolism. Alveolar hemorrhage with abundant hemosiderin-laden histiocytes was observed.

## Discussion

Our patient was a 62-year-old Japanese man with a 4-week history of dyspnea on exertion. Key findings included hypoxemia, severe pulmonary hypertension, and negative pulmonary angiogram. Recurrence of gastric cancer was confirmed from the results of a bone marrow biopsy; PTTM was considered. He was treated with conservative management, following partial alleviation of respiratory failure, and died 6 weeks after admission. Most patients with PTTM die within a week of dyspnea onset; however, our patient survived for 10 weeks from the day of presentation.

PTTM, a special type of tumor thromboembolism, is found in 3.3% of postmortem cases of carcinoma; of all PTTM cases, 52% are attributable to gastric cancer (especially mucinous or signet ring adenocarcinoma) [1]. Clinical manifestations include progressive dyspnea, cough, hypoxia, and pulmonary hypertension. Chest radiography is typically normal; reported chest CT findings include beading of peripheral subsegmental arteries, interlobular septal thickening [2], and tree-in-bud pattern [3]. Almost all patients with PTTM die within 1 week of the onset of dyspnea [4], and establishing an antemortem diagnosis is a challenge. The rapid progression usually prevents treatment targeting the primary tumor. The prognosis in this case was better than that in the previous reports. In PTTM, tumor emboli in pulmonary vessels may not only damage the endothelium but also activate the coagulation systems and release inflammatory mediators and growth factors, including tissue factor, vascular endothelial growth factor (VEGF) [5], platelet-derived growth factor (PDGF) [6], and osteopontin [7], which induce fibrocellular intimal proliferation. PDGF induces macrophage recruitment and VEGF upregulation in cancer cells [8], which leads to inflammation through angiogenesis and enhanced vascular permeability [9]. Osteopontin not only regulates macrophage recruitment but also modulates the production of pro-inflammatory cytokines by macrophages [10]. Several reports have demonstrated that the cytokines interleukin (IL)-1β, IL-6, and tumor necrosis factor-α, released by activated macrophages, play an important role in the pathogenesis of pulmonary arterial hypertension (PAH) [11]. Moreover, inflammatory stimuli including that from IL-1β have been shown to exacerbate hypoxic vasoconstriction in a mouse model of PAH [12]. In PTTM, corticosteroid therapy may help control local inflammation in the pulmonary microvasculature and improve hemodynamics by alleviation of pulmonary vasoconstriction [13]. Our experience with this case suggests that the control of local inflammation with corticosteroid therapy may have helped improve the prognosis, although the time from the onset of pulmonary symptoms to death ranged from 1 day to 12 weeks in one series [14].

## Conclusions

PTTM should be considered in the differential diagnosis of patients with cancer who present with severe pulmonary hypertension. In PTTM, local inflammation in the pulmonary microvasculature may contribute to pulmonary hypertension, and control of inflammation with corticosteroids may help improve the prognosis.

## Abbreviations

CT: Computed tomography
CTPA: Computed tomography pulmonary angiogram
IL: Interleukin
PAH: Pulmonary arterial hypertension
PDGF: Platelet-derived growth factor
PTTM: Pulmonary tumor thrombotic microangiopathy
spO_2_: Blood oxygen saturation
VEGF: Vascular endothelial growth factor
